# Small molecule studies: the fourth wave of muscle research

**DOI:** 10.1007/s10974-019-09526-w

**Published:** 2019-06-21

**Authors:** Steven Marston

**Affiliations:** Cardiovascular Division, National Heart and Lung Institute, Imperial Centre for Translational and Experimental Medicine, Imperial College London, Hammersmith Campus, Du Cane Road, London, W12 0NN UK

**Keywords:** Myosin, Actin, Troponin, Tropomyosin, Contraction, Small molecules, Regulation

## Abstract

The study of muscle and contractility is an unusual scientific endeavour since it has from the start been focussed on one problem—What makes muscle work?—and yet has needed a vast range of different approaches and techniques to study it. Its uniqueness lies in the fundamental fascination of a large scale molecular machine that converts chemical energy into mechanical energy at ambient temperature and with high efficiency that is also controlled by an exquisitely intricate yet utterly reliable regulatory system and is an essential component of animal life. The investigation of muscle is as innovative as any other field of research. As soon as one approach appears to be played out another comes along. It is instructive to consider this as a series of waves of novel and heightened activity starting in the 1950s. The thesis of this article is that we are approaching the fourth wave with the recent rise of interest in small molecules as research tools and possible therapies for muscle diseases.

## Introduction

The study of muscle and contractility is an unusual scientific endeavour since it has from the start been focussed on one problem—What makes muscle work?—and yet has needed a vast range of different approaches and techniques to study it. Its uniqueness lies in the fundamental fascination of a large scale molecular machine that converts chemical energy into mechanical energy at ambient temperature and with high efficiency that is also controlled by an exquisitely intricate yet utterly reliable regulatory system and is an essential component of animal life. The focused nature of muscle research has given rise to a community of muscle scientists that have stuck together for over half a century. The European Muscle Society and its conferences are a prime example of this.

The investigation of muscle is as innovative as any other field of research. As soon as one approach appears to be played out another comes along. It is instructive to consider this as a series of waves of novel and heightened activity. The thesis of this article is that we are approaching the fourth wave with the recent rise of interest of small molecules as research tools and possible therapies for muscle diseases.

The fundamentals of muscle biochemistry were laid down by the 1940s. Actin and myosin were identified as the contractile proteins and ATP hydrolysis by myosin was shown to be the fuel for contractility. These are admirably summarised in the book “Chemistry of Muscle contraction” (Szent-Gyorgyi 1951) published by Albert Szent-Gyorgyi in 1951, based on the work of the Medical Institute of Szeged, a facsimile of which was presented to attendees at the last European Muscle Society conference in Budapest (Kellermeyer 2018). At this time no one knew how these proteins could work to produce muscle contraction.

The first wave of modern muscle research was the structural and mechanical investigations of the 50s and 60s that established the structures of thick and thin filaments, the sliding filament mechanism and the role of myosin crossbridges. The key investigators were Andrew Huxley, who inferred the sliding filament mechanism from light microscopy studies and later inferred the role of crossbridges as independent force-generating units from mechanical studies, especially the length-tension relationship (Huxley 1957a; Huxley and Niedergerke 1954) and Hugh Huxley (no relation). Huxley’s pioneering electron microscope studies, along with Jean Hanson and others directly visualised thick and thin filaments, the sliding filament mechanism and later on, the existence of myosin crossbridges, detectable in rigor but not relaxed muscle (Huxley and Hanson 1954; Huxley 1957b; Reedy et al. 1965). By the early 60s we had a good idea of how the muscle molecular motor was assembled into the contractile apparatus, but we could only guess at how it worked in the absence of mechanistic studies.

The second wave of muscle research was the rise of the biochemists. Myosin is an ATPase that also moves and creates force. Our understanding of how this works was advanced by the kinetic studies pioneered in the labs of Ed Taylor, David Trentham and Evan Eisenberg (Bagshaw et al. 1974; Bagshaw and Trentham 1973; Bagshaw and Trentham 1974; Eisenberg and Moos 1968; Lymn and Taylor 1971).This was complemented by analysis of mechanical transients, notably by Huxley and Simmons (Huxley and Simmons 1971). Very soon the idea of the crossbridge cyle, uniting enzymic and structural pathways was established and became the bedrock of all subsequent studies on muscle contractility. At the same time the question of muscle regulation was also tackled. Ca^2+^ was established as the controlling factor of the contractile apparatus and troponin and tropomyosin were isolated and their mode of action was determined (Bremel and Weber 1972; Ebashi and Endo 1968; Lehman 2017). Later on smooth muscle myosin regulation by phosphorylation (Bremel 1974; Sobieszek and Small 1976) and PKA phosphorylation of TnI in cardiac muscle (England 1976; Ray and England 1976; Solaro et al. 1976) were added to the knowledge base, the protein were sequenced and the structure of g-actin (Kabsch et al. 1990), Myosin S-1 (Rayment et al. 1993a, b), tropomyosin and troponin C (Caspar et al. 1969; Herzberg and James 1985) was determined by X-ray crystallography. Many novel techniques were tried to improve upon our understanding of mechanism: Electron Spin Resonance, Fluorescence polarisation, ATP analogues etc. However, by the early 80s there was a feeling that muscle studies had gone as far as they could. This was changed by the genetic revolution.

The third wave of muscle research was kicked off by advances in molecular biology. This allowed the proteins of muscle to all be sequenced rapidly for the first time. Bacterial expression and genetic manipulation enabled many of the proteins of muscle (but not actin or myosin) to be produced in quantity and purity not previously possible and the introduction of site directed mutagenesis enabled structure–function analysis for many of the key components of muscle. Later on the introduction of transgenic technology enabled these mutations to be incorporated into a living organism (mouse, drosophila) for physiological study. However, the greatest boost to muscle research was the discovery of mutations in contractile proteins that caused human diseases (Geisterfer-Lowrance et al. 1990; Nowak et al. 1999; Seidman and Seidman 2001). This quickly changed muscle research from a largely academic study to a clinically relevant field of endeavour with the accompanying expansion of interest by a wider range of researchers and of funding, thus making muscle research a larger and more active research topic.

A great deal of research has gone into working out how mutations in the contractile machinery can cause inherited diseases, notably Hypertrophic cardiomyopathy, dilated cardiomyopathy and congenital skeletal muscle myopathies. These studies have energised basic studies of muscle structure and biochemistry to a level well beyond that achieved previously. The focus has switched from the basic mechanisms of muscle contraction towards the subtle modulation of contractility by second messengers that modulate phosphorylation of muscle proteins and the effects of disease-related mutations that are generally quite limited (Marston 2016, 2018, 2011; Hershberger et al. 2013; Force et al. 2010; Chang and Potter 2005; Seidman and Seidman 2001; Spudich 2014). This in turn has needed new techniques capable of studying these subtle changes, that often involve intrinsically disordered parts of regulatory proteins (Hwang et al. 2014; Zamora et al. 2016).

The exposure of basic scientists to the translational potential of their work has introduced a growing field of study of small molecules that could modulate contractility in a therapeutically useful way. This is now a significant field of study: at the Alpbach Muscle and Motors meeting in 2016, 1/104 abstracts were devoted to small molecules whilst at the 2019 Alpbach meeting it had grown to 13/86. Both the 2018 European Muscle Conference and the 2019 Alpbach meeting held sessions devoted to small molecules for the first time. Thus it is my hypothesis that this will be the next wave of innovative research, which I have termed, the fourth wave.

The impetus of the fourth wave is to find small molecules that have potential for reversing the abnormalities of the contractile apparatus associated with muscle diseases, primarily in skeletal and cardiac muscle. At the same time these studies have provided new insights into muscle regulatory mechanisms.

In general, muscle disease can be classified as hypocontractile or hypercontractile (gain-of-function). Hypocontractile disease include congenital skeletal muscle myopathies which are generally due to mutations (nemaline myopathy, congenital fibre-type disproportion etc.) and failing heart. Heart failure is actually the term for a large group of unrelated defects in which the heart does not produce enough work to sustain normal contraction or does not have sufficient reserve to respond to stimulus in exercise. It is unlikely that any single agent could be found that can correct all forms of heart failure but a significant fraction of heart failure (for instance, dilated cardiomyopathy) is caused by specific mutations and in many cases the mechanism has been defined (Marston 2011; Memo et al. 2013; Messer and Marston 2014). In these cases, a target for small molecule action could be defined. Currently most research is being directed towards myosin activators, Ca^2+^-sensitisers and recouplers.

The hypercontractile muscle diseases are nearly all due to mutations. In skeletal muscle gain-of-function mutations are associated with distal arthrogryposis or the more extreme ‘stiff child’ syndrome (Jain et al. 2012; Donkervoort et al. 2015; Memo and Marston 2013; Robinson et al. 2007). In cardiac muscle hypertrophic cardiomyopathy is the classic gain-of-function syndrome and this is an attractive target for potential small molecule treatments (Spudich 2014). Current research focuses on Ca^2+^-desensitisers and myosin inhibitors.

## Ca^2+^-sensitisers

The Ca^2+^-sensitising drugs that act upon troponin are the classic muscle activators that have been studied in heart muscle for decades.

Bepridil, Levosimedan and EMD57033 have been extensively studied for their ability to increase Ca^2+^-affinity for cardiac troponin C and increase Ca^2+^-sensitivity of regulated thin filaments in many assay systems. They have also been investigated in structural assays by NMR in particular (Hwang and Sykes 2015; Robertson et al. 2010). As potential muscle activators to treat heart failure—cardiotonics—they have been notably unsuccessful and are only prescribed for acute support of the heart post operatively or in toxic shock in a hospital setting. The reasons for this are related to their basic biochemical properties. As a class many Ca^2+^-sensitisers are not specific enough and have significant phosphodiesterase inhibiting activity as well as Ca^2+^-sensitising activity, which can have deleterious side effects. A compound, currently in development, AMG 594, appears to be a cardiac-specific Ca^2+^-sensitiser without any off target action and may thus be a safer drug than current compounds (Cytokinetics 2019). However, there are additional problems inherent in the concept of Ca^2+^-sensitisation. In many aspects sensitisers like bepridil and EMD57033 mimic the effect of HCM mutations: they blunt or abolish the modulation of Ca^2+^-sensitivity by phosphorylation of TnI in response to adrenergic stimulation (Papadaki et al. 2015) and they enhance the probability of arrhythmia (Baudenbacher et al. 2008; Huke and Knollmann 2010; Rowlands et al. 2017). Clinical experience has shown that although these compounds can boost cardiac output and patent wellbeing in the short term, they do not address the underlying defect in the failing heart. Chronic treatment leads to a worsening of symptoms and increased mortality.

Recently a series of Ca^2+^-sensitisers specific to fast skeletal muscle have been developed as potential treatment of various congenital myopathies. Since skeletal muscle can regenerate, unlike cardiac, it is thought that muscle activation will promote muscle growth. Interestingly these compounds have been found to have potential in myopathies that are neural in origin as well as those due to skeletal muscle defects. There are reports that CK-2066260, CK-2017357 (now called Tirasemtiv) and CK-2127107 (now called Reldesemtiv) are fast skeletal muscle specific Ca^2+^-sensitisers (Collibee et al. 2018; Hwee et al. 2017; Hwee et al. 2015) and that they can alleviate the symptoms of nebulin-related nemaline myopathy (de Winter et al. 2013), Amyotrophic lateral sclerosis (Hwee et al. 2014) and spinal muscular atrophy. These small molecules may be of great research interest in understanding the mechanisms of muscle growth and atrophy.

## Ca^2+^-desensitisers and recouplers

There is a small group of small molecules that act on troponin to reduce Ca^2+^-sensitivity; these would have potential in the treatment of hypercontractile diseases such as HCM (Spudich, 2014; Semsarian et al. 2002; Tadano et al. 2010). The most widely investigated small molecule is epigallocatechin-3-gallate (EGCG) (Papadaki et al. 2015; Tadano et al. 2010) but desensitisation can also be observed in vitro using Nebivolol, W-7, epicatechin gallate (ECG) and Silybin A (Frampton and Orchard 1992; Sheehan et al. 2018; Stücker et al. 2017; Zeitz et al. 2000). Interestingly EGCG and Nebivolol have been shown to be cardiac specific and so presumably interact with the unique phosphorylation dependent modulation of Ca^2+^ sensitivity. Moreover EGCG was shown to restore cardiac output in isolated working hearts by improving diastolic dysfunction caused by increased myofilament Ca^2+^ sensitivity in a mouse model of hypertrophic cardiomyopathy, thus demonstrating the principle that Ca^2+^ desensitisation has potential for treatment of HCM. Although current desensitisers are interesting research molecules, with the exception of Nebivolol they have numerous known off target effects that would render them useless as treatments for cardiac disease (Ingólfsson et al. 2014).

Uncoupling is a common abnormality in cardiac troponin that is closely related to abnormal Ca^2+^-sensitivity. In many cases of DCM and both inherited and non-mutation linked HCM it has been found that myofilament Ca^2+^-sensitivity is independent of the level of troponin I phosphorylation, leading to a blunted response to adrenergic stimulation and loss of cardiac reserve (Memo et al. 2013; Messer et al. 2016; Messer et al. 2017; Messer and Marston 2014; Vikhorev et al. 2014, Biesiadecki et al. 2007; Dvornikov et al. 2016; Wilkinson et al. 2015). Remarkably a number of small molecules have been shown to restore the phosphorylation-dependent Ca^2+^ sensitivity shift of uncoupled HCM and DCM mutant troponin and tropomyosin to normal (Papadaki et al. 2015; Sheehan et al. 2018). Recouplers found to date have a range of chemical structures that include EGCG (in addition to its desensitising property), Silybin B, resveratrol, pterostilbene and novobiocin. This is an area where structure–function relationships can pinpoint the key molecular requirements and where these functional probes could shed important light on the mechanism of the phosphorylation-dependent Ca^2+^-sensitivity shift. The combination of desensitisation with recoupling activity, as shown by EGCG is in principle an ideal functional profile for the treatment of HCM.

## Myosin activators and inhibitors

There is currently much interest in small molecules that are specific cardiac muscle myosin activators or inhibitors. Omecamtiv Mecarbil is a myosin activator that is now in phase 3 clinical trials as a treatment for heart failure and Mavacamten is a myosin inhibitor that has just started phase 2 trials as a treatment for HCM. Both these molecules were found using high throughput screening protocols that have not been published in the scientific press, however since the compounds have become available for researchers their investigation has given new insights into muscle regulation in the thick filament.

Omecamtiv Mecarbil was developed on the principle that a specific cardiac myosin activator would avoid many of the disadvantages of Ca^2+^-sensitisers including increased heart rate, increased oxygen demand, decreased efficiency and enhanced probability of arrhythmias (Malik et al. 2011; Malik and Morgan 2011; Teerlink et al. 2011). It increases twitch tension magnitude and lifetime and thus increases work output without compromising efficiency. The mechanism of action is particularly interesting. In early studies the key effect was proposed to be a 4-fold acceleration of the phosphate release step of the crossbridge cycle which was proposed to increase the proportion of the crossbridge cycle in the force generating states, i.e. an increased duty cycle. A number of basic observations are at variance with such a direct interpretation, notably that Omecamtiv Mecarbil actually inhibits actin filament movement in the in vitro motility assay and increases Ca^2+^-sensitivity. (Liu et al. 2015; Swenson et al. 2017). Moreveover, the effect of Omecamtiv Mecarbil is biphasic with inhibition of force production in skinned fibres above 1 µM (Nagy et al. 2015). Woody et al’s study has solved the problem by demonstrating an indirect mechanism (Woody et al. 2018). Optical trap measurements showed that Omecamtiv Mecarbil reduced the size of the crossbridge stroke from 5 nm to zero at 10 µM. This accounts for the inhibitory action of Omecamtiv Mecarbil since attached non-moving crossbridges would act as a brake. Since Omecamtiv Mecarbil prolongs myosin attachment duration, both in steady state assays and in the optical trap, crossbridges with Omecamtiv Mecarbil bound also act as cooperative activators of the thin filament by the well known mechanism (Bremel et al. 1972; Lehrer and Geeves 1998). This accounts for the activating effect of Omecamtiv Mecarbil and also the enhanced Ca^2+^-sensitivity. Thus the effect of Omecamtiv Mecarbil is ultimately on the recruitment of crossbridges rather than the dynamics of the crossbridge cycle.

Another mechanism for modulating thick filament activity has recently been found based on the interacting heads motif of myosin. Current thinking about the actin–myosin interaction is that the thick filament exists in two states; these have for a long time been described as ordered (SRX) and disordered (DRX) thick filament states and their transition appeared to be regulated by phosphorylation. The “ordered” state corresponds to a structure in which the myosin heads, instead of pointing away from the thick filament towards actin are folded backwards and inwards to form the compact interacting heads motif close to the thick filament shaft in a position where interaction with actin is not possible, referred to as the interacting heads motif (IHM) (Trivedi et al. 2017). The presence of this ‘super-relaxed’ (SRX) state in striated muscle including human cardiac muscle has now been demonstrated, using single ATP turnover kinetics of relaxed skinned muscle which shows a fast and a slow process with t_1/2_ of 14.3 and 224 secs respectively in human cardiac muscle. In normal cardiac muscle the proportion of heads in the “super-relaxed state” was 27.6 ± 0.7% (McNamara et al. 2014; McNamara et al. 2017). It has been established that HCM mutations in thick filament proteins often affect the super-relaxed state. The effects of destabilising the SRX would be incomplete relaxation and more myosin heads available leading to hypercontractility—the key abnormalities of HCM. The higher proportion of myosin heads would also act upon the thin filament cooperatively to promote the open state, which would cause an increase in the apparent Ca^2+^-sensitivity of muscle activation as observed. DCM causing mutations may conversely stabilise the SRX, thus SRX has become a target for small molecules that can inhibit or activate myosin (Trivedi et al. 2017).

A study by Cooke et al. found that piperine was able to destabilise the SRX in skeletal muscle and it was proposed that this could be a treatment for obesity and diabetes, since it would increase muscle energy expenditure at rest (Nogara et al. 2016). Recently it was shown that Mavacamten acts by stabilising the SRX in cardiac muscle, thus accounting for its effectiveness as an antagonist to the effects of HCM mutations (Anderson et al. 2018). Blebbistatin and its analogues have long been used as myosin inhibitors in vitro and these have now also been shown to work via stabilising the SRX (Wilson et al. 2014; Kampourakis et al. 2017).

## Conclusions

**Fig. 1:**
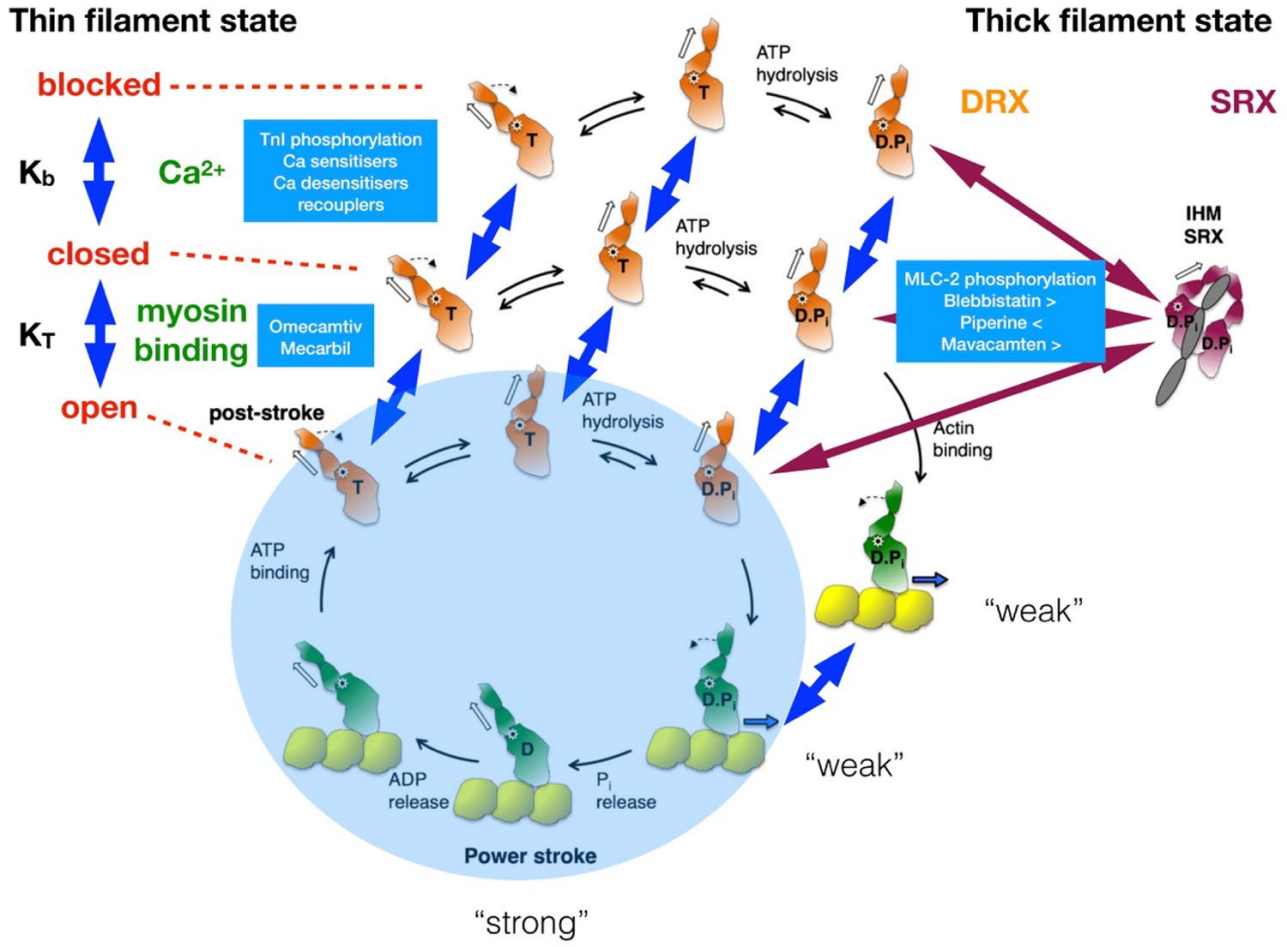
The crossbridge cycle and its regulation by troponin-tropomyosin and by the SRX/DRX equilibrium. The chemomechanical crossbridge cycle is represented in the blue circle. The availability of actin-binding sites is controlled by troponin–tropomyosin (top left). Ca^2+^ controls the equilibrium between blocked (no myosin binding) and closed (weak myosin binding) states. Myosin heads cooperatively regulate the closed-open equilibrium. Only the open state may participate in the crossbridge cycle. Small molecules that interact with each transition are shown. The availability of myosin heads is controlled by the SRX-DRX equilibrium; only the DRX state can enter the crossbridge cycle. Small molecules and physiological regulators that modulate the transition are shown. Diagram modified from Spudich (2019, Fig. 5). (Color figure online)

As research into small molecules that act on contractile proteins gathers pace a pattern is beginning to emerge. Few, if any, of the small molecules so far studied act directly on the crossbridge cycle but they do interfere with modulation of contractility by Ca^2+^ and troponin/tropomyosin, the DRX–SRX equilibrium and on phosphorylation mechanisms (see Fig. 1). Overall these small molecules seem to work by controlling the recruitment of actin sites or myosin heads that undergo the full contractile cycle rather than affecting the crossbridge cycle itself (Spudich 2014).

In conclusion, this fourth wave of muscle research promises exciting discoveries and a new impetus to the field of muscle research. Already studies with small molecules have clarified several aspects of muscle regulation and their use as probes of mechanisms has great potential. Basic research goes hand-in-hand with the corresponding potential of specific small molecules to modify and correct the abnormalities of contractility in muscle diseases and thus be of therapeutic value. The new disciplines of combinational synthetic chemistry, computational chemistry and high throughput assays are now being harnessed to muscle research. The scene is set for a new wave of experiments and insight into how muscle works.
